# Effect of frozen storage conditions on antithrombin protein C and protein S activity assay stability

**DOI:** 10.1186/s12959-024-00640-5

**Published:** 2024-07-29

**Authors:** Houmei Feng, Danyu Song, Qiang Xu, Xiaohui Cai, Jianru Liu, Yang Zhang, Zhou Zhou

**Affiliations:** 1 Department of Laboratory Medicine, Nanfang Hospital, Southern Medical University, Guangzhou, 510515 China; 2 Department of Laboratory Medicine, National Center for Cardiovascular Diseases and Fuwai Hospital, Peking Union Medical College and Chinese Academy of Medical Sciences, No. 167 Beilishi Road, Xicheng District, Beijing, 100037 China

**Keywords:** Antithrombin, Frozen plasma, Protein C, Protein S, Storage, Thrombophilia

## Abstract

**Background:**

Inherited antithrombin, protein C, and protein S deficiency increase the risk of venous thromboembolism. The presence of defects can be identified by clinical laboratory assays. In most Chinese clinical laboratories, the screening tests for antithrombin, protein C, and protein S deficiency are their activity assays. Ensuring appropriate pre-analytical storage conditions for activity tests is essential. This study aimed to assess the effects of storage conditions on antithrombin, protein C, and protein S activity in frozen plasma.

**Methods:**

We collected the remaining plasma of 29 patients. The baseline of antithrombin, protein C, and protein S activity values were tested within 4 h. Then, each sample was sub-packaged into 4 EP tubes, and was stored at -20 °C for 3 days, -20 °C for 7 days, -80 °C for 3 days, and − 80 °C for 7 days, respectively. After thawing, samples were tested by two systems.

**Results:**

The percentage deviation of antithrombin and protein C activity assay was<10% compared with the initial values. Protein S activity showed a significant reduction in frozen plasma, with a deviation > 10%. Some samples, initially within the normal range, were classified as abnormal after freezing storage.

**Conclusions:**

Our study indicated that antithrombin and protein C remain stable when stored at -20 °C or -80 °C in a week. We argued that Protein S activity is not stable in frozen plasma. The use of frozen-thawed plasma for PS activity assay may result in overdiagnosis of protein S deficiency.

## Introduction

Inherited antithrombin (AT), protein C (PC) and protein S (PS) deficiency increase the risk of venous thromboembolism (VTE) [1]. Clinical laboratory assays play a crucial role in identifying these defects [2, 3]. Based on the recommendations for clinical laboratory testing for AT, PC, and PS deficiency of ISTH (International Society for Thrombosis and Haemostasis), they recommended chromogenic activity assay as the initial test for AT and PC deficiency [4, 5]; and the initial assay for PS deficiency should be free PS antigen assay [6]. Because PS activity assays have more potential to generate falsely decreased PS values than antigenic-based assays [7, 8]. However, the reasons for the false decrease are unidentified. In the majority of Chinese clinical laboratories, the screening tests for inherited AT, PC, and PS deficiency are their activity assays. In routine clinical work, AT, PC, and PS activity assays were performed every two or three days. Notably, we have observed that PS activity values tend to fall under the low-normal range after undergoing frozen storage, exhibiting a larger coefficient of variation compared to AT and PC activity assays. Despite these observations, ISTH recommends that samples for PS testing can be safely frozen below − 20 °C for a duration of up to 2 weeks or up to 18 months below − 70 °C [6]. To assess the effects of time and temperature on AT, PC, and PS activity in frozen plasma, and to put forward the best scheme of clinical laboratory testing, we designed this research.

## Methods

### Preparation of samples and conditions for storage

We selected 29 patients with normal AT, PC, PS activity value. The patients who pregnant or undergoing anticoagulation treatment were excluded. We collected the remaining sodium-citrate-anticoagulated (0.109 M trisodium citrate, concentration 3.2%) plasma of 30 first-admitted patients routine coagulation tests. Samples from each patient were centrifuged (10 min, 3000 × g) to obtain platelet poor plasma (PPP) within 2 h after blood sampling.

**Fig. 1:**
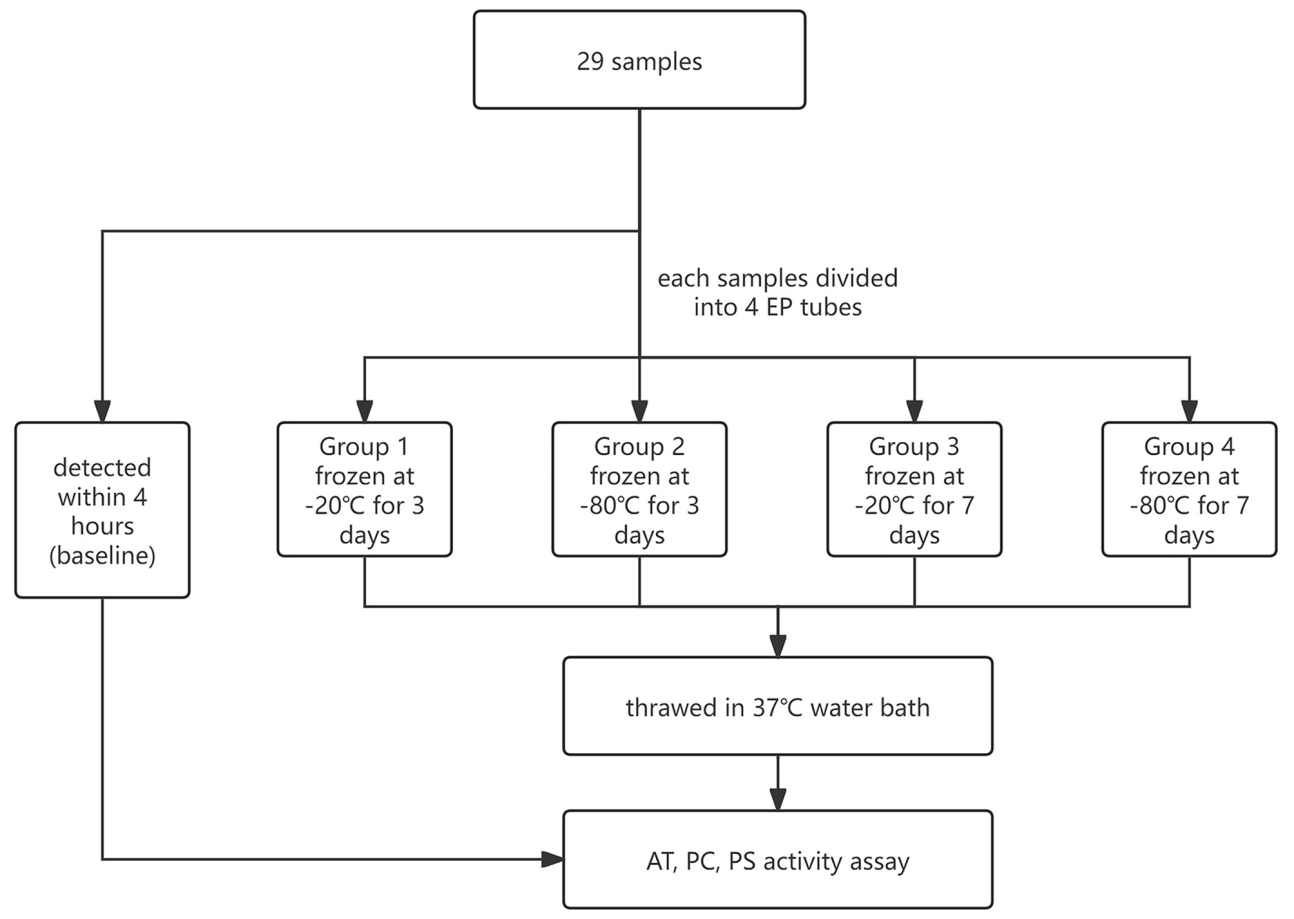
Flow chart depicting the experimental design

According to the recommendation of ISTH, AT, PC, and PS activity assay were completed within 4 h of collection [4–6]. This value served as a baseline. Test procedure compliance the guideline of Clinical and Laboratory Standards Institute (CLSI) [9]. Then, each plasma was subpackaged into 4 EP tubes. As shown in Fig. 1, Two aliquots were frozen at -20 °C and the other two aliquots were frozen at -80 °C at a stable temperature (two fridges without auto-defrost mechanism). After 3 days and 7 days, frozen plasma specimens had been rapidly thawed at 37 °C, then gently mixed and tested immediately [10].

### Analysers and reagents

All the samples and aliquots were tested for AT activity (Chromogenic assay), PC (Chromogenic assay) activity, and PS activity (Clotting assay) using two systems and reagents. Using ACL TOP 750^®^ (Instrumentation Laboratory, Bedford, MA, USA)and STA Compact Max^®^(Diagnostica Stago, Asnières-sur-Seine, France)coagulation instrument. The reagents and assay methods used in this study are listed in Table 1. 

**Table 1:** List of assay methods and reagents used in this study

	Platforms used in analysis	Method	Reagent	LOT	CVa (Normal Control)	Reference range(%)
Antithrombin activity assay	ACL TOP 750, IL	Chromogenic assay	HemosIL^®^ Liquid Antithrombin	N0897936	4.3	83–128
	STA-R Max, Stago	Chromogenic assay	STACHROM^®^ ATIII	257105	5.3	80–120
Protein C activity assay	ACL TOP 750, IL	Chromogenic assay	HemosIL^®^ Protein C	N0302595	2.8	70–140
	STA-R Max, Stago	Chromogenic assay	STACHROM^®^ PROTEIN C	257266	5.1	70–130
Protein S activity assay	ACL TOP 750, IL	Clotting assay	HemosIL^®^ Protein S Activity	N1199930	5.9	63.5–149
	STA-R Max, Stago	Clotting assay	STACLOT^®^ PROTEIN S	255927	7.5	Male:77–143 Female:55–123

### Statistical analysis

Categorical variables are reported as numbers and percentages. The Shapiro-Wilk test was used to verify whether the data were normally distributed. A *p* value of 0.05 was used to determine the level of significance. Continuous data is expressed as mean ± standard deviation.

Percentage deviation of the analyte value is calculated by subtracting the baseline value (T0) and the value for a specific time (Tx, Day3, Day7) using the following formula:

$$\text{Percentage}\:\text{deviation}=\left[\frac{\left({\text{T}}_{\text{x}}-{\text{T}}_{0}\right)}{{\text{T}}_{0}}\right]\times100\text{\%}$$

The changes occurring on storage were expressed as a percentage of the initial value:

$$\text{Percentage}\:\text{of}\:\text{initial}\:\text{value}\left(\text{\%}\right)=\text{percentage}\:\text{deviation}+100$$

Furthermore, we used the total change limit (TCL) to evaluate their stability. The TCL was also calculated using the formula [11]:

$$\text{TCL}=\sqrt{{\left(2.77\times\text{CVa}\right)}^{2}+{\left(0.5\times\text{CVb}\right)}^{2}}$$

CVa is the analytical imprecision of the assay. The CVa was obtained from the average of the accumulated QC values for each analyte over six months. The CVa of this study has been listed in Table 1. CVb is the within-subject variation. The CVb of each analyte was taken from the databases on biological variation. We found the CVb of AT, PC, and PS activity assay from the databases were 5.2, 5.6, and 5.8. When the percentage deviation (± SD) was found to be higher than the TCL, the difference was judged to be significant and unacceptable.

Differences between the baseline and frozen-thawed results were also analyzed by repeated measures ANOVA. Comparisons between time points were made using the Bonferroni correction. The statistical analysis was using Statistical Package for Social Sciences (SPSS) for Windows. GraphPad Prism (GraphPad) was used to plot these results into graphs.

### Ethical considerations

This study was approved by the Ethics Committees of Fuwai Hospital, Peking Union Medical College, and Chinese Academy of Medical Sciences.

## Results

### Deviation of AT PC PS activity assay

**Fig. 2:**
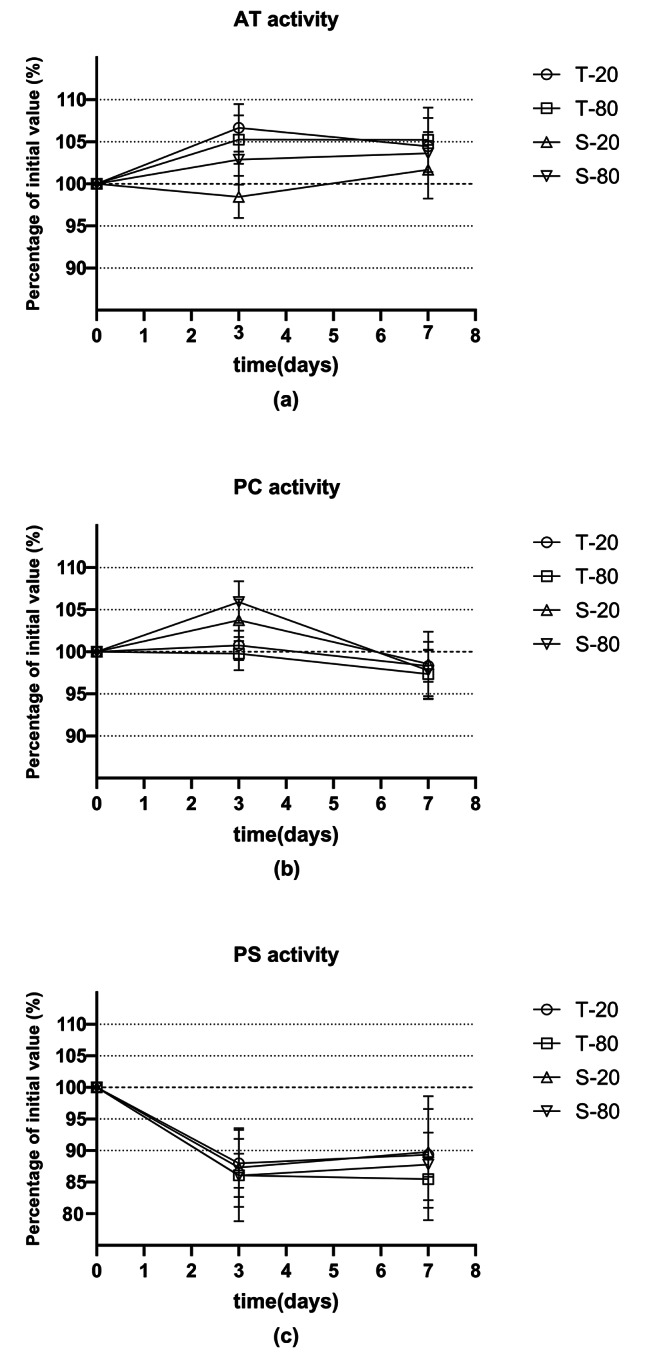
Percentage of initial value of AT activity (**a**), PC activity (**b**), PS activity (**c**). T-20:stored at -20 °C fridge and was detected by ACL TOP 750 coagulation instrument; T-80: stored at -80 °C fridge and was detected by ACL TOP 750 coagulation instrument; S-20: stored at -20 °C fridge and was detected by STA Compact Max coagulation instrument; S-80: stored at -80 °C fridge and was detected by STA Compact Max coagulation instrument

As shown in Table 2 and Fig. 2a, compared with the initial values, it was clear that the percentage deviation of AT activity assay was <±10%. Compare between two detection system, STA Compact Max^®^ showed less deviation than ACL TOP 750. According to PC activity, the percentage of deviation was all<±10% compared with the initial values (Fig. 2b; Table 2). We considered it acceptable as a clinically relevant difference. Thus, AT activity and PC activity were stable when stored at -20 °C or -80 °C in a week. PS activity showed a prominent reduction at -20 °C or -80 °C in 3 days and 7 days. The percentage deviation of PS activity assay was significant > 10% (Fig. 2C; Table 2) from the baseline values. We argued that PS activity was not stable when using the frozen plasma.

**Table 2:** Stability of AT, PC and PS activity at -20 °C and − 80 °C for 3 days and 7 days

Parameter	T0	TCL	-20 °C				-80 °C			
			D3	deviation(%)	D7	deviation(%)	D3	deviation(%)	D7	deviation(%)
**(A)ACL TOP 750**										
AT(%)	105.97 ± 12.91	± 12.19	112.90^***^	6.65	110.66^***^	4.46	111.45^***^	5.27	111.45^***^	5.25
PC(%)	122.38 ± 22.23	± 8.25	123.24	0.77	120.17^***^	-1.70	122.07	-0.22	119.03^***^	-2.64
PS(%)	113.29 ± 21.45	± 16.6	99.37^***^	-12.01	101.09^***^	-10.66	97.47^***^	-13.93	96.71^***^	-14.51
**(B)STA Compact Max**										
AT(%)	102.55 ± 9.11	± 14.91	101.03^**^	-1.54	105.38^***^	2.88	104.24^*^	1.69	106.28^***^	3.64
PC(%)	115.62 ± 22.18	± 14.4	119.90^***^	3.76	113.90	-1.44	122.48^***^	5.92	112.79^**^	-2.22
PS(%)	88.38 ± 18.49	± 20.98	77.07^***^	-12.67	78.90^***^	-10.21	75.59^***^	-13.97	77.10^***^	-12.23

Samples were tested by coagulation detecting platform ACL TOP 750 (A) and STA Compact Max (B)

^*^p < 0.05; ^**^p < 0.01; ^***^p < 0.001

### Evaluate by total change limit

We also used the total change limit (TCL) to evaluate their stability. According to the formulas and CVa, CVb in this study, then we obtained the percentage deviation and TCL (Table 2). From Table 2 it can be seen that the percentage deviation of AT and PC activity was much lower than their TCL, either 3 or 7 days, either stored at -20 °C or -80 °C. AT and PC activity were considered quite stable in the frozen plasma. As can be seen, each percentage deviation of PS activity was > 10%, however, it did not exceed the corresponding TCL. So if we evaluated the stability of PS activity in this way (TCL), the change of PS activity was considered acceptable.

### Evaluate by repeated measures ANOVA

Furthermore, differences between the baseline and frozen-thawed results were analyzed by repeated measures ANOVA. Although AT and PC activity were considered stable, as shown in Table 2, the values of frozen samples had statistical differences at *P* < 0.05 compared to the baseline value. PS activity value showed a more remarkable statistical difference. Compared to the baseline value, the results of frozen plasma declined significantly.

### Classification changes in the PS activity assay

**Fig. 3:**
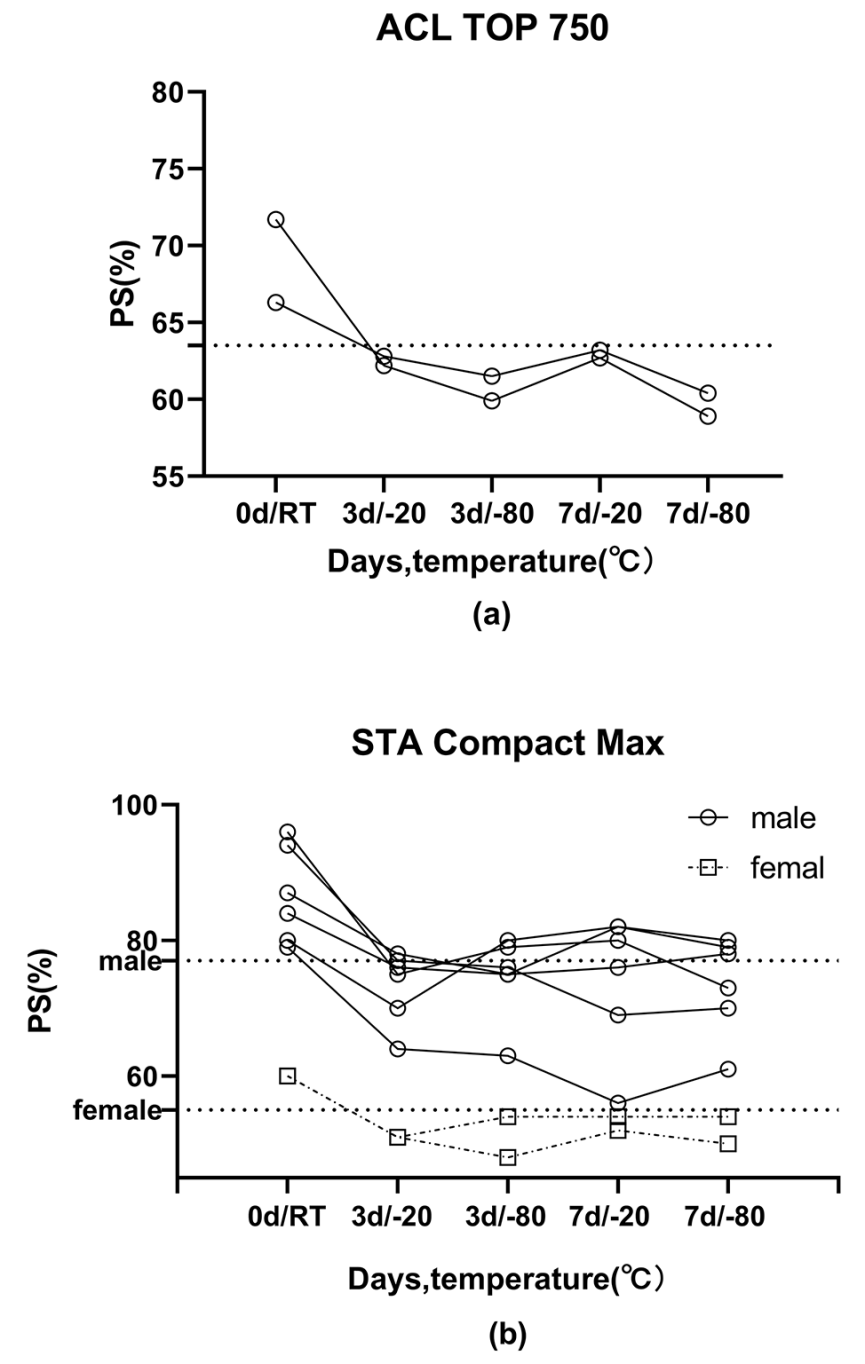
Patients with classification changes in frozen plasma. Samples were tested by coagulation detecting platform ACL TOP 750 (**a**) and STA Compact Max (**b**). Dashed lines showed the lower limit of reference range (ACL TOP 750:63.5%, STA Compact Max: 77% in male and 55% in female)

To analyze the impact of permanent reduction of PS activity in frozen plasma on patient clinical decisions, we filtered the patients with classification changes whose baseline values were within the reference range but frozen plasma values were below the lower limit of the reference range. Using ACL TOP 750, 2 (6.9%) patients have classification changes in PS values, regardless of storage conditions. The details are shown in Fig. 3a; Table 3. In terms of using STA Compact Max, the results were generally lower than those by ACL TOP 750. Two patients who had classification changes by ACL TOP 750, were all below the lower limit of reference range at baseline when tested by STA Compact Max. Another two patients were below the lower limit in both baseline and frozen plasma values, and the results were normal by ACL TOP 750. Furthermore, 8(27.6%) patients occurred classification changes. The details are shown in Fig. 3b; Table 3. In this group of patients, freezing storage may result in overdiagnosis of PS deficiency.

**Table 3:** Details of patients with abnormal classification in protein S activity assay

	Sex	ACL TOP 750(%) (Normal:63.5–149%)					STA Compact Max(%) (Normal: male:77–143, female:55–123%)				
		0d/RT	3d/-20 °C	3d/-80 °C	7d/-20 °C	7d/-80 °C	0d/RT	3d/-20 °C	3d/-80 °C	7d/-20 °C	7d/-80 °C
1	M	66.3	62.8^*^	61.5^*^	63.2^*^	60.4^*^	59^*^	48^*^	46^*^	50^*^	46^*^
2	F	81.1	75.1	69.4	71.7	69.7	60	51^*^	48^*^	52^*^	50^*^
3	M	109.2	94.5	94.3	98	92.3	84	76^*^	75^*^	82	79
4	M	118.4	103.1	100.9	103.6	100.3	96	75^*^	79	80	73^*^
5	M	116.3	100.4	92.4	96.9	94.1	80	70^*^	80	82	80
6	M	90.8	82.1	78.2	83.2	78.1	76^*^	69^*^	65^*^	72^*^	69^*^
7	F	81.1	69.5	66.5	69.1	67.1	60	51^*^	54^*^	54^*^	54^*^
8	M	117.1	97.4	92.8	96.1	94.3	87	78	75^*^	76^*^	78
9	F	71.7	62.2^*^	59.9^*^	62.7^*^	58.9^*^	46^*^	43^*^	43^*^	45^*^	43^*^
10	M	78.3	72.5	70.3	72.5	72.1	55^*^	46^*^	58^*^	58^*^	57^*^
11	M	116.1	101	100.6	105	97.1	94	77	76^*^	69^*^	70^*^
12	M	88.1	77.8	73	78.7	76.7	79	64^*^	63^*^	56^*^	61^*^

^*^value below the lower limit of reference

## Discussion

In the recommendations of ISTH, frozen samples for AT testing can be stored for up to 24 months at ≤-24 °C [4]. In PC tests, frozen samples can be stored below − 20 °C for up to 2 weeks or up to 18 months below − 70 °C [5]. The same as PC tests, samples for PS tests can be frozen below − 20 °C for up to 2 weeks or up to 18 months below − 70 °C [6]. However, the recommendation does not indicate whether this stability is for antigen assay or activity assays. In part of clinical laboratories in China, the frequency of AT, PC, and PS activity tests was to be 2–3 times a week. So, the plasma will be packaged and frozen first for centralized testing.

In our study, four evaluation methods were used to assess the effect of storage time and temperature on the activity of antithrombin, protein C, and protein S.

According to our research, the percentage deviation of AT and PC activity assay was<10%. It can be seen that the activity of AT and PC was stable when stored at -20 °C or -80 °C for at least a week. However, the activity of PS was unstable in frozen plasma. The results showed significant negative bias, the percentage deviations of PS activity assay were all > 10%.

TCL had been calculated to assess the stability of common biochemical analytes [11–13], which takes into account the acceptable imprecision based on the analytical imprecision of the assay and the within-subject variation. In our study, interestingly, although PS activity was considered unstable, they did not exceed the TCL. We were considering it was associated with the analytical imprecision of PS assay. From a research by Mark T. Cunningham et al. [14], the all-method accuracy and precision of PS assays were lower than AT, and PC assays in laboratories in United States. The causes of lower accuracy and precision of PS assay, especially PS activity assay may be related to its reagents and detection method.

When we used repeated measures ANOVA to find whether there was a statistical difference between the baseline and frozen-thawed results, most values of AT and PC frozen samples had statistic differences at *P* < 0.05 compared to baseline. The results of PS activity were of course the same. We considered that this method was not suitable for the stability evaluation of coagulation parameters.

Furthermore, 6.9% (2/29) tests using ACL TOP 750 and 27.6% (8/29) tests using STA Compact Max had classification changes in frozen plasma. Their baseline PS values were within the reference range, but were below the limit in some or all of the storage conditions. In this case, if PS activity is postponed, and samples are frozen after acceptance by laboratory in advance will result in a false-reduction of PS activity and overdiagnosis of PS deficiency.

The recommendations for clinical laboratory testing for PS deficiency of ISTH has emphasized that activity assays may result in overdiagnosis of PS deficiency, as PS activity assays have a higher potential to generate falsely decreased PS values than the antigenic-based assays [6]. While the PS activity assay is sensitive in identifying all types of inherited PS deficiency, both qualitative and quantitative. However, activity assay has a specificity of only 40–70% [6, 15, 16]. Therefore, despite the possibility of the free PS antigen assay missing the diagnosis of type II PS deficiencies, the recommended initial assay for PS deficiency should be the free PS antigen assay rather than PS activity assays. Currently, there is a limited availability of registration certificates for free PS antigen reagents in the Chinese market, and the screening test for protein S deficiency is replaced by PS activity assays.

In previous studies, the effect on the results varied due to differences in freezing conditions and duration. In a former study by Woodhams et al [17], PS assay can achieve a ≤ ± 5% variation for 8 and 6 months when stored at -74 °C and − 24 °C, respectively. In the study of Zander [18], Free protein S showed slightly decrease when stored at ≤-20 °C, ≤-80 °C, ≤-130 °C for 7 days. Gupta et al. study confirmed that AT, PC and free PS all declined significantly after a fortnight freezing at -25 °C [19]. After a 24 h freezing, only free PS observed a slightly decline [19]. Data comparing differences between PS activity assays and PS antigen assays are lacking. A previous study revealed that PS activity and free PS increased during the first week after freezing, moreover PS activity decreased after 2 month [20]. Our study confirmed that PS activity decreased significantly after only 3 days of freezing storage. In conclusion, PS is labile in cryopreservation, especially the activity assay. To obtain the most accurate results possible, the test should be performed as soon as possible after blood collection by venipuncture, and it is inappropriate to use frozen specimens for the test.

There were also several limitations in our study. Firstly, our sample size is small and the duration of storage is short in our study. However the storage duration in the design can cover the needs of clinical laboratories. Secondly, we weren’t able to test free PS antigen in parallel to analyze this reduction whether qualitative or quantitative.

## Conclusions

Our study indicated that AT and PC are stable when stored at -20 °C or -80 °C in a week. PS activity showed a prominent reduction at -20 °C or -80 °C in 3 days and 7days. The percentage deviation of PS activity assay was significant > 10% from the baseline values in frozen plasma. Some samples that are normal at baseline are classified to be abnormal after freezing storage. We argued that Protein S activity was not stable when using the frozen plasma. Our results can guide the laboratory’s clinical practice to obtain more accurate test results in AT, PC, PS activity assays.

## Data Availability

No datasets were generated or analysed during the current study.

## Abbreviations

AT: antithrombin
PC: protein C
PS: protein S
VTE: venous thromboembolism
ISTH: International Society for Thrombosis and Haemostasis
CLSI: Clinical and Laboratory Standards Institute
TCL: total change limit
CV: coefficient of variance
QC: quality control
SD: standard deviation
